# Supplementary material for: Hydrolysis Mechanism of Carbamate Methomyl by a Novel Esterase PestE: A QM/MM Approach
Source: Int J Mol Sci. 2022 Dec 27;24(1):433. doi: 10.3390/ijms24010433 (PMC9820155; doi:10.3390/ijms24010433)
Supplement: Supplementary file 1 [file ijms-24-00433-s001.zip › ijms-2102623-supplementary.pdf]

Supplementary data for

Hydrolysis Mechanism of Carbamate Methomyl by a Novel Esterase PestE：A QM/MM approach

Zijian Wang, Qingzhu Zhang*, Guoqiang Wang*, Wenxing Wang, Qiao Wang

Big Data Research Center for Ecology and Environment, Environment Research Institute, Shandong University, Qingdao 266003, P. R. China

Keywords: methomyl; hydrolysis; esterase; biotransformation; nucleophilic attack; quantum mechanics/molecular mechanics; molecular dynamics

---

*Corresponding authors. E-mail: zqz@sdu.edu.cn, 202188900012@sdu.edu.cn

Table S1. Key bond distances (in Å) at the M06-2X//6-31G(d,p)//CHARMM27 level during the whole reactions in 4 snapshots.

Table S2. The force field of Methomyl generated by SwissParam.

Figure S1. TTcluster was applied to RMSD and cluster analysis of 30 ns MD simulations performed with CHARMM. (a) The heatmap of the possible cluster number of the system is saved by the distance matrix; (b) Clustering dendrogram of five clusters of the system; (c) The distribution of clusters in dynamic simulation; (d) 2D projection plot of the relative distances among five clusters based on the RMSD of representative frames.

Figure S2. Root-mean-square deviations (RMSD) of the backbone for molecular dynamic simulation involved in the system.

Cartesian coordinates of QM regions of all computed species during the reaction.

Table S1. Key bond distances (in Å) at the M06-2X//6-31G(d,p)//CHARMM27 level during the whole reactions in 4 snapshots.

Distances (Å)		SN2	SN3	SN4	SN5
**d(C^1-O^1)**	R	2.68	2.50	2.53	2.48
	TS1	1.76	1.79	1.73	1.82
	IM	1.51	1.52	1.52	1.53
	TS2	1.38	1.38	1.38	1.38
	P	1.34	1.32	1.34	1.33
**d(O^1-H^1)**	R	0.98	0.99	0.98	0.99
	TS1	1.41	1.34	1.38	1.36
	IM	1.62	1.52	1.63	1.52
	TS2	2.23	2.19	2.18	2.15
	P	2.57	2.39	2.28	2.28
**d(H^1-N^1)**	R	1.92	1.71	1.82	1.70
	TS1	1.15	1.18	1.16	1.15
	IM	1.07	1.09	1.06	1.08
	TS2	1.20	1.17	1.22	1.19
	P	1.80	1.72	1.77	1.67
**d(H^1-O^3)**	R	3.21	3.36	3.37	3.40
	TS1	3.39	3.34	3.37	3.44
	IM	3.37	3.29	3.49	3.43
	TS2	1.31	1.36	1.29	1.33
	P	0.99	1.00	0.99	1.01
**d(C^1-O^3)**	R	1.34	1.34	1.34	1.34
	TS1	1.39	1.38	1.39	1.38
	IM	1.43	1.42	1.42	1.42
	TS2	1.88	1.87	1.87	1.89
	P	2.60	2.67	2.66	2.62

Table S2. The force field of Methomyl generated by SwissParam.

```
RESI LIG     0.000
GROUP
ATOM C       CR       0.3001
ATOM N       NC=O -0.7301
ATOM C1      C=O     0.7800
ATOM O       O=C   -0.5700
ATOM H       HNCO    0.3700
ATOM O1      OR     -0.0870
ATOM N1      N=C   -0.5130
ATOM C2      C=O     0.5300
ATOM C3      CR       0.0610
ATOM S       SMMF -0.3710
ATOM C4      CR       0.2300
ATOM H1      HCMM    0.0000
ATOM H2      HCMM    0.0000
ATOM H3      HCMM    0.0000
ATOM H4      HCMM    0.0000
ATOM H5      HCMM    0.0000
ATOM H6      HCMM    0.0000
ATOM H7      HCMM    0.0000
ATOM H8      HCMM    0.0000
ATOM H9      HCMM -0.0000
BOND H3      C
BOND H2      C
BOND C       H1
BOND C       N
BOND O       C1
BOND N       C1
BOND N       H
BOND C1      O1
BOND O1      N1
BOND N1      C2
BOND H6      C3
BOND C2      C3
BOND C2      S
BOND C3      H5
BOND C3      H4
BOND S       C4
BOND H9      C4
BOND C4      H7
```

```
BOND C4   H8
IMPH C    H3   N    H2
IMPH C    H3   N    H1
IMPH N    C1   C    H
IMPH C1   O1   N    O
IMPH C2   S    N1   C3
IMPH C3   H6   C2   H5
IMPH C3   H6   C2   H4
IMPH C4   H9   S    H7
IMPH C4   H9   S    H8
IC C    N    C1   O     1.45   119.67   -8.97   126.18   1.20
IC C    N    C1   O1    1.45   119.67  171.99   108.18   1.38
IC N    C1   O1   N1    1.36   108.18  -87.31   112.58   1.40
IC C1   N    C    H1    1.36   119.67 -180.00   109.47   1.07
IC C1   N    C    H2    1.36   119.67   59.98   109.47   1.07
IC C1   N    C    H3    1.36   119.67  -60.00   109.48   1.07
IC C1   O1   N1   C2    1.38   112.58 -157.79   110.72   1.27
IC O    C1   N    H     1.20   126.18 -169.36   117.23   1.00
IC O    C1   O1   N1    1.20   125.64   93.65   112.58   1.40
IC H    N    C    H1    1.00   120.09  -20.17   109.47   1.07
IC H    N    C    H2    1.00   120.09 -140.19   109.47   1.07
IC H    N    C    H3    1.00   120.09   99.82   109.48   1.07
IC H    N    C1   O1    1.00   117.23   11.60   108.18   1.38
IC O1   N1   C2   C3    1.40   110.72  179.97   116.79   1.50
IC O1   N1   C2   S     1.40   110.72    0.08   121.75   1.77
IC N1   C2   C3   H4    1.27   116.79 -179.99   109.46   1.07
IC N1   C2   C3   H5    1.27   116.79   59.98   109.49   1.07
IC N1   C2   C3   H6    1.27   116.79  -60.02   109.47   1.07
IC N1   C2   S    C4    1.27   121.75  171.24   102.06   1.81
IC C2   S    C4   H7    1.77   102.06  180.00   109.44   1.07
IC C2   S    C4   H8    1.77   102.06   60.01   109.46   1.07
IC C2   S    C4   H9    1.77   102.06  -60.03   109.46   1.07
IC C3   C2   S    C4    1.50   121.46   -8.66   102.06   1.81
IC S    C2   C3   H4    1.77   121.46   -0.10   109.46   1.07
IC S    C2   C3   H5    1.77   121.46 -120.12   109.49   1.07
IC S    C2   C3   H6    1.77   121.46  119.87   109.47   1.07
IC H3   N    *C   H2    0.00     0.00  120.00     0.00   0.00
IC H3   N    *C   H1    0.00     0.00 -120.00     0.00   0.00
IC C1   C    *N   H     0.00     0.00  180.00     0.00   0.00
IC O1   N    *C1  O     0.00     0.00  180.00     0.00   0.00
IC S    N1   *C2  C3    0.00     0.00  180.00     0.00   0.00
IC H6   C2   *C3  H5    0.00     0.00  120.00     0.00   0.00
IC H6   C2   *C3  H4    0.00     0.00 -120.00     0.00   0.00
IC H9   S    *C4  H7    0.00     0.00  120.00     0.00   0.00
```

IC H9 S *C4 H8 0.00 0.00 -120.00 0.00 0.00

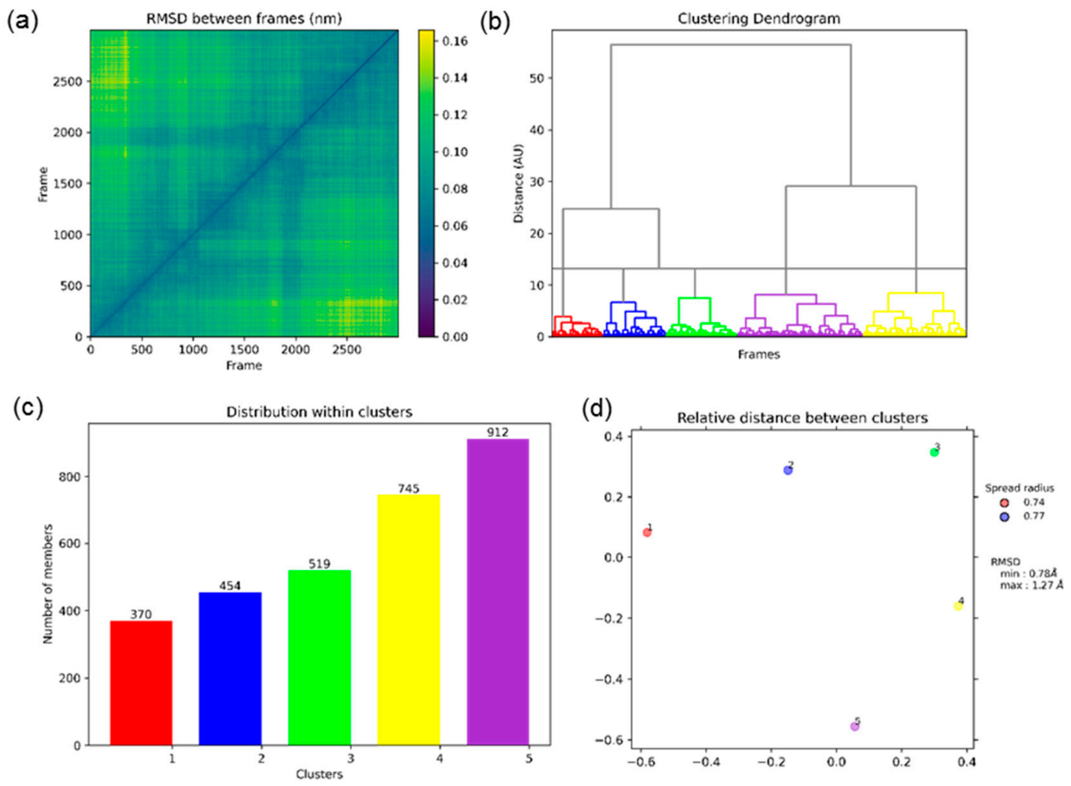

Figure S1. TTcluster was applied to RMSD and cluster analysis of 30 ns MD simulations performed with CHARMM. (a) The heatmap of the possible cluster number of the system is saved by the distance matrix; (b) Clustering dendrogram of five clusters of the system; (c) The distribution of clusters in dynamic simulation; (d) 2D projection plot of the relative distances among five clusters based on the RMSD of representative frames.

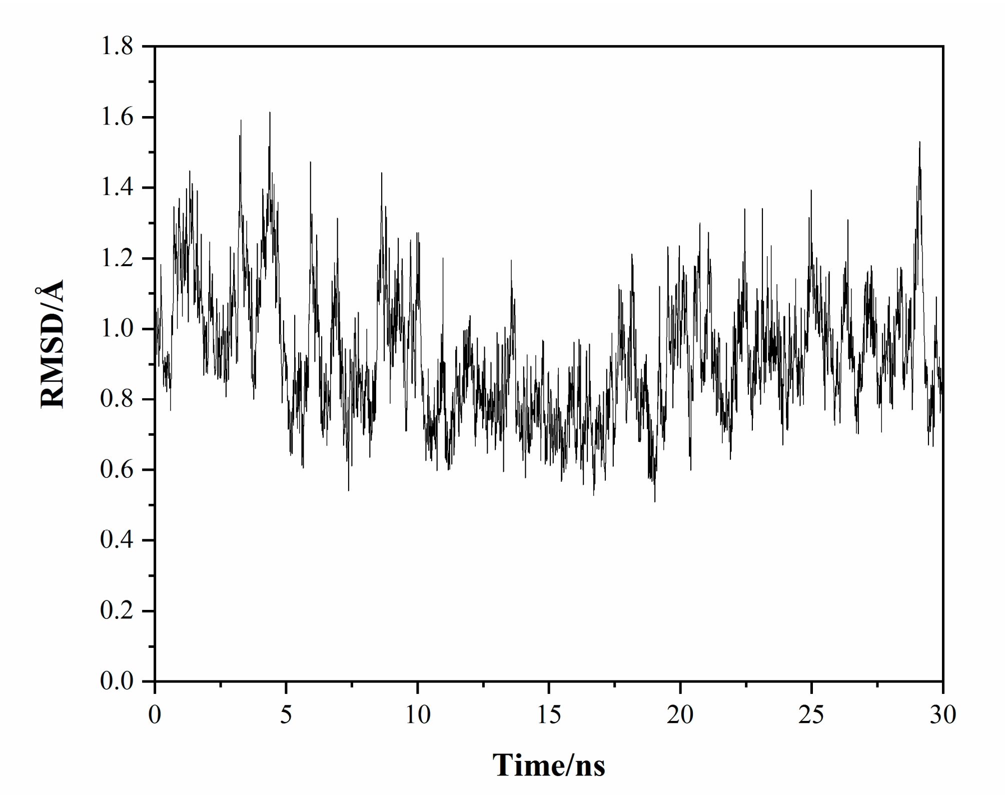

Figure S2. Root-mean-square deviations (RMSD) of the backbone for molecular dynamic simulation involved in the system.

Cartesian coordinates of QM regions of all computed species during the reaction.

Reactant(SN1)

N 5.189023 -1.356398 -4.901213
H 5.409537 -0.862375 -4.043487
C 6.037059 -1.055082 -6.047076
H 5.719502 -1.597275 -6.941087
H 7.031098 -1.461682 -5.806036
C 6.326061 0.398711 -6.502249
O 6.809017 0.511422 -7.616039
N 2.776532 0.730490 0.874738
H 2.801753 0.524707 1.871101
C 3.953982 1.443047 0.356176
H 4.265987 2.059174 1.203580
C 5.162589 0.546894 0.045166
H 5.273204 -0.150663 0.888558
H 4.983712 -0.053046 -0.860586
O 6.297249 1.364490 -0.085698
H 7.063997 0.866178 0.263723
C 3.524765 2.515201 -0.702916
O 2.943924 3.477960 -0.221150
N 11.834381 -2.264366 5.516402
H 12.749786 -1.987975 5.155820
C 10.648548 -2.012033 4.703367
H 9.815873 -1.694670 5.348990
C 10.996346 -0.822525 3.770305
H 11.406629 -0.006009 4.369008
H 11.780041 -1.164619 3.087412
N 8.918730 0.568743 3.472872
H 9.019963 1.052869 4.395728
C 9.824382 -0.336442 2.973582
C 7.963249 0.763384 2.542727
H 7.127759 1.439302 2.677008
N 8.186223 0.023848 1.463865
C 9.351736 -0.661394 1.726088
H 9.776180 -1.362044 1.021154
C 10.186019 -3.185395 3.781073
O 11.044227 -3.944231 3.374259
C 8.051262 -0.578697 -2.457099
N 7.956956 0.867288 -2.395030
C 6.785132 1.449371 -2.611344
O 5.767965 0.879540 -3.031484
H 8.650441 1.419676 -1.910377
O 6.685971 2.777165 -2.460815
N 7.805240 3.391309 -1.906927
C 7.750098 4.644603 -2.164123
C 8.849091 5.501300 -1.614039
S 6.396145 5.323596 -3.083451
C 7.125442 6.888399 -3.626996
H 9.033245 -0.886717 -2.103477
H 7.926291 -0.927856 -3.484469
H 7.280578 -1.041506 -1.837948
H 8.443391 6.255709 -0.935902
H 9.382861 6.025682 -2.409462
H 9.544810 4.868924 -1.062214
H 7.456523 7.504420 -2.792102
H 6.336414 7.417416 -4.162061
H 7.951984 6.720443 -4.316181
h 4.204908 -1.452560 -5.050354
h 6.188944 1.271447 -5.863815
h 2.297968 0.036555 0.336778
h 3.627751 2.437735 -1.785255
h 11.831112 -3.017077 6.174724
h 9.141841 -3.338202 3.508292

TS1(SN1)

N	5.254457	-1.392970	-4.728505
H	5.419657	-0.860891	-3.876523
C	6.134666	-1.065654	-5.843709
H	5.873029	-1.616429	-6.751727
H	7.134249	-1.433391	-5.563196
C	6.391043	0.397396	-6.282469
O	6.813885	0.526906	-7.420056
N	2.904998	0.685628	0.854350
H	2.970572	0.479153	1.848371
C	4.069174	1.358052	0.243372
H	4.475078	1.950742	1.071036
C	5.183596	0.371525	-0.185932
H	5.308684	-0.321740	0.664663
H	4.857220	-0.224291	-1.045499
O	6.410063	0.981381	-0.460449
H	7.364784	0.606743	0.454461
C	3.572401	2.462159	-0.762870
O	2.994464	3.396882	-0.227448
N	11.675822	-2.146973	5.385919
H	12.592733	-1.880179	5.018486
C	10.488069	-1.933235	4.557272
H	9.651104	-1.621845	5.199451
C	10.808837	-0.758356	3.589272
H	11.257742	0.059607	4.159503
H	11.555887	-1.115484	2.874121
N	8.782355	0.719697	3.393204
H	8.931157	1.139190	4.348167
C	9.621957	-0.222381	2.835259
C	7.841342	1.059222	2.509365
H	7.047770	1.774140	2.677630
N	8.026220	0.370405	1.385675
C	9.134750	-0.436774	1.570295
H	9.492817	-1.101445	0.799741
C	10.061051	-3.131904	3.651080
O	10.950961	-3.843197	3.228924
C	8.151038	-0.548532	-2.444039
N	8.011930	0.890009	-2.339986
C	6.710711	1.344906	-2.140194
O	5.759164	0.848025	-2.818488
H	8.671945	1.353022	-1.728431
O	6.622343	2.730393	-2.068172
N	7.744958	3.357457	-1.571081
C	7.725856	4.586322	-1.926679
C	8.846597	5.470963	-1.469080
S	6.368135	5.213296	-2.889256
C	7.102643	6.738839	-3.534041
H	9.191426	-0.824551	-2.264108
H	7.885085	-0.887662	-3.447787
H	7.512467	-1.083663	-1.730243
H	8.473568	6.289438	-0.847472
H	9.377072	5.918761	-2.312932
H	9.546085	4.873021	-0.883228
H	7.454967	7.401037	-2.743290
H	6.310395	7.247507	-4.084968
H	7.916121	6.525235	-4.227516
h	4.281550	-1.507043	-4.929529
h	6.288096	1.252983	-5.615057
h	2.347459	0.018243	0.360673
h	3.633516	2.396610	-1.849164
h	11.670029	-2.892464	6.052388
h	9.020424	-3.351899	3.412828

IM(SN1)

N	5.240386 -1.365697 -4.771540
H	5.386908 -0.806790 -3.931073
C	6.121349 -1.029761 -5.882684
H	5.866717 -1.574037 -6.795901
H	7.120685 -1.397337 -5.598748
C	6.382656 0.435864 -6.308125
O	6.773611 0.576308 -7.456743
N	3.027657 0.533823 0.778466
H	3.091684 0.413395 1.782761
C	4.151566 1.191606 0.088814
H	4.624453 1.778626 0.885134
C	5.188908 0.138944 -0.378490
H	5.304716 -0.546572 0.471265
H	4.802528 -0.437466 -1.221798
O	6.482971 0.611990 -0.680011
H	7.531016 0.590471 0.500410
C	3.593039 2.308800 -0.878789
O	3.007491 3.206599 -0.293638
N	11.646622 -2.155460 5.343975
H	12.568664 -1.865455 5.008737
C	10.474016 -1.911189 4.509173
H	9.632399 -1.585154 5.138326
C	10.854305 -0.742169 3.561234
H	11.333805 0.050946 4.141496
H	11.590860 -1.125635 2.849357
N	8.845222 0.758276 3.387430
H	8.977237 1.152635 4.358377
C	9.694869 -0.163373 2.811550
C	7.911503 1.123806 2.512818
H	7.103325 1.819476 2.687822
N	8.126436 0.464713 1.377665
C	9.229770 -0.346422 1.537920
H	9.579815 -0.987756 0.745262
C	10.037925 -3.096414 3.583930
O	10.933137 -3.767620 3.112742
C	8.315062 -0.485397 -2.661219
N	8.036557 0.915486 -2.438102
C	6.684255 1.195046 -2.060906
O	5.766712 0.790937 -2.880788
H	8.675518 1.337684 -1.772904
O	6.533419 2.600620 -1.851539
N	7.658628 3.227599 -1.363422
C	7.657267 4.449157 -1.743007
C	8.800842 5.322749 -1.326341
S	6.306011 5.070154 -2.718246
C	7.040369 6.601269 -3.354870
H	9.331180 -0.597550 -3.044649
H	7.622876 -0.872446 -3.411505
H	8.215274 -1.115979 -1.763804
H	8.468182 6.167184 -0.718261
H	9.324979 5.731961 -2.192551
H	9.497661 4.720929 -0.742043
H	7.375185 7.267751 -2.559934
H	6.253101 7.103191 -3.918336
H	7.866028 6.392012 -4.035184
h	4.270405 -1.504592 -4.971079
h	6.308506 1.279402 -5.621821
h	2.391964 -0.090094 0.323941
h	3.631909 2.278523 -1.967660
h	11.631256 -2.901567 6.009603
h	8.995793 -3.335289 3.371877

TS2(SN1)

N 5.187761 -1.146806 -4.830026
H 5.303249 -0.571214 -4.004589
C 6.142823 -0.893835 -5.900084
H 5.897916 -1.444506 -6.811616
H 7.109895 -1.296871 -5.562774
C 6.442902 0.558082 -6.339784
O 6.782982 0.698692 -7.503537
N 3.426701 -0.365380 0.766627
H 3.294703 -0.094390 1.734700
C 4.410993 0.391536 -0.024305
H 5.085708 0.819906 0.725905
C 5.241793 -0.642597 -0.839665
H 5.196468 -1.566139 -0.262940
H 4.785597 -0.832782 -1.813245
O 6.632440 -0.407659 -1.016631
H 7.462151 1.057544 0.458662
C 3.779954 1.656522 -0.737120
O 3.071695 2.331053 -0.009208
N 11.569650 -2.127253 5.321097
H 12.488643 -1.844333 4.973681
C 10.387028 -1.883244 4.498919
H 9.549473 -1.570877 5.140000
C 10.731653 -0.702407 3.552925
H 11.214654 0.088509 4.133892
H 11.455336 -1.070334 2.820148
N 8.759614 0.836600 3.447821
H 8.928576 1.208653 4.417500
C 9.552483 -0.110089 2.837859
C 7.834006 1.259614 2.583563
H 7.074405 1.999669 2.784860
N 7.975781 0.616702 1.433537
C 9.043092 -0.244902 1.570479
H 9.368165 -0.885693 0.765814
C 9.956181 -3.059765 3.565317
O 10.850020 -3.715319 3.068879
C 8.673275 -0.345847 -3.344843
N 8.377742 0.535450 -2.218926
C 7.009695 0.576265 -1.919717
O 6.199357 0.877477 -2.828679
H 8.915791 0.312057 -1.389619
O 7.071921 1.827290 -0.554438
N 8.152051 2.692122 -0.540491
C 7.962194 3.765516 -1.205575
C 9.050846 4.795856 -1.178290
S 6.445591 4.056412 -2.069379
C 6.760950 5.673848 -2.825106
H 9.733964 -0.597303 -3.328228
H 8.452035 0.169524 -4.280852
H 8.098039 -1.281926 -3.318397
H 8.703220 5.721202 -0.711514
H 9.405874 5.041705 -2.180067
H 9.873996 4.395758 -0.589359
H 6.936044 6.458621 -2.088990
H 5.853550 5.917057 -3.381094
H 7.591334 5.642261 -3.531365
h 4.238720 -1.351617 -5.069495
h 6.438240 1.404306 -5.652797
h 2.599311 -0.750295 0.357681
h 3.874062 1.890458 -1.797537
h 11.555219 -2.868976 5.991627
h 8.912795 -3.303338 3.365107

Product(SN1)

N	5.116754 -1.455030 -5.022298
H	5.260658 -0.934122 -4.167864
C	6.081065 -1.163477 -6.075289
H	5.847711 -1.705782 -6.994480
H	7.039041 -1.575457 -5.719852
C	6.410220 0.293589 -6.485818
O	6.776074 0.455725 -7.636778
N	3.366968 -0.421879 0.767074
H	3.270685 -0.160752 1.742643
C	4.393682 0.245269 -0.038119
H	5.105686 0.644396 0.694222
C	5.127254 -0.874742 -0.817975
H	5.080535 -1.758869 -0.184345
H	4.626166 -1.105910 -1.759721
O	6.517509 -0.685800 -1.056901
H	7.520126 1.079167 0.286727
C	3.847934 1.520481 -0.793347
O	3.191167 2.262792 -0.085543
N	11.799078 -2.276060 5.525876
H	12.715935 -2.023701 5.150167
C	10.618571 -2.022049 4.710034
H	9.785490 -1.706783 5.355644
C	10.978625 -0.827373 3.791995
H	11.415380 -0.031038 4.400480
H	11.744153 -1.171116 3.088885
N	8.948081 0.630302 3.570887
H	9.072046 1.072730 4.511592
C	9.804559 -0.297246 3.033094
C	7.999293 0.905933 2.655688
H	7.197336 1.614639 2.819536
N	8.175388 0.193738 1.553853
C	9.304580 -0.559580 1.781384
H	9.701000 -1.243682 1.043994
C	10.155122 -3.181139 3.769080
O	11.009530 -3.943678 3.361046
C	8.942868 0.483254 -3.385039
N	8.229672 -0.192022 -2.331149
C	6.911705 -0.101713 -2.188526
O	6.129477 0.385741 -3.017962
H	8.736031 -0.574423 -1.544762
O	7.223196 1.803911 -0.341965
N	8.316828 2.631292 -0.393604
C	8.128363 3.621971 -1.175949
C	9.192571 4.670999 -1.299698
S	6.603034 3.748028 -2.078501
C	6.817979 5.322901 -2.952836
H	9.933521 0.743742 -3.013644
H	8.411935 1.398179 -3.655166
H	9.049062 -0.148829 -4.270974
H	8.819557 5.656633 -1.005908
H	9.566912 4.749084 -2.323433
H	10.013551 4.404647 -0.634719
H	6.883977 6.170068 -2.268887
H	5.920597 5.442421 -3.563042
H	7.684109 5.320157 -3.616405
h	4.149100 -1.538256 -5.260395
h	6.410687 1.126639 -5.782899
h	2.529890 -0.789460 0.361912
h	3.941283 1.715843 -1.861610
h	11.791259 -3.032562 6.179800
h	9.112093 -3.318724 3.484078

Reactant(SN2)

N	4.672046	-0.523235	-4.819268
H	5.060180	-0.279768	-3.916822
C	5.627584	-0.550170	-5.915473
H	5.142372	-0.850886	-6.844569
H	6.469899	-1.241163	-5.760634
C	6.259490	0.816130	-6.257180
O	6.846392	0.871159	-7.325628
N	2.787519	0.878285	1.177844
H	2.747567	0.743662	2.186332
C	3.969047	1.603446	0.675530
H	4.266656	2.211851	1.531892
C	5.211371	0.757740	0.355189
H	5.317185	0.001329	1.141510
H	5.073751	0.227687	-0.600832
O	6.318108	1.609114	0.300671
H	7.106234	1.070012	0.509532
C	3.551802	2.680575	-0.380313
O	2.945056	3.633234	0.092422
N	11.896973	-2.215004	5.701225
H	12.792334	-1.862592	5.361187
C	10.709433	-2.012286	4.884012
H	9.861972	-1.711623	5.519345
C	11.026880	-0.833286	3.925660
H	11.434242	0.002836	4.498232
H	11.800635	-1.178189	3.233741
N	8.854179	0.415257	3.675992
H	8.908527	0.877237	4.613531
C	9.829987	-0.391242	3.141937
C	7.898942	0.591718	2.742971
H	7.024899	1.211492	2.905274
N	8.187319	-0.063213	1.626198
C	9.398792	-0.675538	1.870530
H	9.886862	-1.301516	1.136226
C	10.284321	-3.202889	3.967262
O	11.163890	-3.925916	3.543577
C	7.970856	-0.625140	-2.295152
N	7.935442	0.812358	-2.086474
C	6.841752	1.517024	-2.323400
O	5.771551	1.074138	-2.760812
H	8.713598	1.301397	-1.667521
O	6.872839	2.847549	-2.153701
N	8.025995	3.354348	-1.567445
C	8.049242	4.619336	-1.764109
C	9.176099	5.394644	-1.157608
S	6.757696	5.409868	-2.684186
C	7.580516	6.963218	-3.113431
H	6.977360	-1.039907	-2.124841
H	8.668075	-1.069705	-1.586578
H	8.286087	-0.869264	-3.312418
H	9.824062	4.704613	-0.618318
H	8.789302	6.139266	-0.458189
H	9.758294	5.925323	-1.913017
H	8.492679	6.789772	-3.681953
H	7.793695	7.584572	-2.244422
H	6.882094	7.489279	-3.763854
h	4.002265	-1.262205	-4.746547
h	6.220191	1.697801	-5.617509
h	2.338613	0.141791	0.671849
h	3.688769	2.622237	-1.460083
h	11.933694	-2.963952	6.362814
h	9.240911	-3.383590	3.708978

TS1(SN2)

N 4.601498 -0.525076 -4.638355
H 4.938590 -0.221874 -3.729488
C 5.639905 -0.600997 -5.654362
H 5.236364 -0.946178 -6.606466
H 6.471755 -1.276762 -5.397843
C 6.291393 0.752496 -5.992568
O 6.822371 0.820033 -7.091420
N 2.879259 0.759916 1.155946
H 2.894934 0.612550 2.161123
C 4.038282 1.438933 0.545731
H 4.464174 2.002954 1.382005
C 5.140271 0.456792 0.073896
H 5.246757 -0.288363 0.878445
H 4.783650 -0.059422 -0.831833
O 6.380437 1.036572 -0.174111
H 7.384249 0.541814 0.680401
C 3.564376 2.575077 -0.434117
O 2.967821 3.490407 0.116242
N 11.728173 -2.130231 5.580088
H 12.628495 -1.789999 5.237117
C 10.544198 -1.950902 4.745724
H 9.694400 -1.648791 5.376702
C 10.857748 -0.784157 3.767002
H 11.298701 0.047376 4.323336
H 11.603258 -1.145635 3.053818
N 8.733802 0.545841 3.617198
H 8.831618 0.939003 4.590832
C 9.658776 -0.281638 3.018768
C 7.784894 0.864458 2.737377
H 6.934610 1.502996 2.930159
N 8.048186 0.271630 1.577764
C 9.218318 -0.450162 1.730220
H 9.661689 -1.019334 0.926427
C 10.149118 -3.163366 3.841794
O 11.060756 -3.816120 3.375833
C 8.045018 -0.577699 -2.354683
N 7.976619 0.847852 -2.082618
C 6.730190 1.402859 -1.861791
O 5.734730 1.007611 -2.542958
H 8.712388 1.260881 -1.529238
O 6.747542 2.791037 -1.770015
N 7.905809 3.330513 -1.255596
C 7.953771 4.570022 -1.568389
C 9.099452 5.392487 -1.064715
S 6.641970 5.289462 -2.528562
C 7.427496 6.838283 -3.037555
H 7.115653 -1.076479 -2.066165
H 8.867925 -1.035304 -1.799966
H 8.210922 -0.772122 -3.417713
H 9.775477 4.741172 -0.510128
H 8.743113 6.184948 -0.400999
H 9.650002 5.872321 -1.876266
H 8.317879 6.661471 -3.640138
H 7.670689 7.487318 -2.195977
H 6.693973 7.339975 -3.669396
h 3.970396 -1.297085 -4.562968
h 6.328289 1.607448 -5.317466
h 2.285318 0.122917 0.664582
h 3.672068 2.561273 -1.518680
h 11.761726 -2.879983 6.240934
h 9.111760 -3.432880 3.643511

IM(SN2)

N	4.573519	-0.392628	-4.680945
H	4.875210	-0.009603	-3.787605
C	5.640184	-0.492935	-5.663553
H	5.263541	-0.865361	-6.616088
H	6.471400	-1.154169	-5.368058
C	6.289054	0.861844	-6.010331
O	6.797553	0.931083	-7.120445
N	2.935430	0.805892	1.053175
H	3.049124	0.612986	2.040719
C	4.057698	1.455201	0.348423
H	4.562695	2.016429	1.143060
C	5.084651	0.411398	-0.168835
H	5.195304	-0.325771	0.635687
H	4.702152	-0.113282	-1.046899
O	6.377483	0.906081	-0.429431
H	7.545988	0.526729	0.624754
C	3.522129	2.604777	-0.597985
O	2.910192	3.483767	-0.008868
N	11.677107	-2.115293	5.560350
H	12.580437	-1.774971	5.227295
C	10.506417	-1.927786	4.705219
H	9.647939	-1.620875	5.322233
C	10.856323	-0.762963	3.738046
H	11.306793	0.056464	4.303364
H	11.602725	-1.142246	3.035602
N	8.770523	0.628628	3.540715
H	8.855360	1.016625	4.519807
C	9.686263	-0.229840	2.968627
C	7.849945	0.973045	2.644891
H	7.005915	1.626650	2.815200
N	8.132807	0.361076	1.498704
C	9.275890	-0.396015	1.671151
H	9.714795	-0.973318	0.872885
C	10.124705	-3.135576	3.785998
O	11.042980	-3.700739	3.229686
C	8.057640	-0.358642	-2.469445
N	7.960393	1.062421	-2.183290
C	6.642532	1.466528	-1.801653
O	5.704463	1.155538	-2.640050
H	8.632887	1.368605	-1.489183
O	6.606493	2.878753	-1.596100
N	7.769176	3.426669	-1.105143
C	7.836482	4.646097	-1.483910
C	8.984490	5.488377	-1.019904
S	6.530508	5.311505	-2.495026
C	7.321818	6.829863	-3.080119
H	7.750156	-1.003838	-1.631578
H	9.084770	-0.604841	-2.743855
H	7.415584	-0.593986	-3.318871
H	9.661803	4.855930	-0.446040
H	8.641210	6.308263	-0.383222
H	9.531179	5.931493	-1.853767
H	8.197203	6.617408	-3.692440
H	7.591768	7.510159	-2.272660
H	6.580954	7.316685	-3.714289
h	3.959572	-1.173972	-4.568954
h	6.342807	1.712712	-5.331213
h	2.281728	0.202555	0.596416
h	3.613774	2.620279	-1.683999
h	11.708742	-2.875513	6.209223
h	9.095275	-3.468382	3.653407

TS2(SN2)

N	4.627639	-0.350973	-4.564749
H	4.915262	-0.013190	-3.651501
C	5.723364	-0.455642	-5.523290
H	5.372169	-0.855790	-6.474583
H	6.546305	-1.105051	-5.183821
C	6.365449	0.892293	-5.906121
O	6.805395	0.957672	-7.044992
N	3.244339	0.272772	1.063710
H	3.298506	0.239753	2.074074
C	4.304887	0.921663	0.286435
H	4.966486	1.366923	1.038999
C	5.122620	-0.185847	-0.459171
H	5.025315	-1.079928	0.153183
H	4.672692	-0.390062	-1.431722
O	6.520857	-0.033423	-0.631840
H	7.693448	1.360046	0.663773
C	3.735026	2.167192	-0.514367
O	3.055525	2.931087	0.149802
N	11.609665	-2.038326	5.586258
H	12.520886	-1.720520	5.247673
C	10.443664	-1.822698	4.728763
H	9.585020	-1.534239	5.352277
C	10.804530	-0.625847	3.807606
H	11.289531	0.146270	4.413012
H	11.537026	-0.986488	3.079544
N	8.786321	0.862672	3.714603
H	8.875906	1.177443	4.712739
C	9.653265	0.001947	3.080032
C	7.923549	1.356046	2.825152
H	7.123127	2.043294	3.048978
N	8.179014	0.847040	1.628749
C	9.253825	-0.005455	1.768259
H	9.657579	-0.567145	0.940428
C	10.061713	-3.001450	3.776027
O	10.980319	-3.599810	3.251572
C	8.491917	-0.380804	-3.026186
N	8.316335	0.613236	-1.968176
C	6.974088	0.816080	-1.621745
O	6.165453	1.175126	-2.504610
H	8.867582	0.400102	-1.143799
O	7.276367	2.128141	-0.316416
N	8.379011	2.946163	-0.463133
C	8.105869	4.044675	-1.052659
C	9.147733	5.122696	-1.059008
S	6.474419	4.353537	-1.674286
C	6.728465	5.897634	-2.593343
H	7.903703	-1.294645	-2.864451
H	9.546885	-0.652394	-3.074771
H	8.214800	0.055197	-3.986313
H	10.081602	4.701093	-0.687471
H	8.840135	5.930506	-0.386347
H	9.302879	5.565712	-2.042929
H	7.393804	5.761302	-3.448090
H	7.099264	6.710786	-1.967361
H	5.742966	6.172883	-2.975076
h	4.009193	-1.134409	-4.503712
h	6.491026	1.743306	-5.236738
h	2.437309	-0.156298	0.658033
h	3.853434	2.322770	-1.586674
h	11.635450	-2.812941	6.218143
h	9.027699	-3.294640	3.594583

Product(SN2)

N 4.401808 -0.406170 -4.778144
H 4.600353 0.058406 -3.900032
C 5.613722 -0.603814 -5.575151
H 5.380033 -1.088871 -6.522548
H 6.382378 -1.211553 -5.068970
C 6.301935 0.716178 -5.966013
O 6.770062 0.773746 -7.091352
N 2.899160 0.641117 1.139057
H 3.027055 0.436082 2.126099
C 4.072442 1.121676 0.389477
H 4.712320 1.576745 1.153483
C 4.840921 -0.088029 -0.206304
H 4.796547 -0.879076 0.543091
H 4.320276 -0.452298 -1.094610
O 6.219018 0.064008 -0.490775
H 8.431126 1.264527 0.036288
C 3.656244 2.330670 -0.531361
O 3.052006 3.219585 0.044444
N 11.915835 -2.168751 5.818597
H 12.826527 -1.865133 5.470800
C 10.759302 -1.926340 4.964468
H 9.905746 -1.600703 5.578370
C 11.175980 -0.751351 4.045007
H 11.553972 0.069515 4.657240
H 12.001792 -1.114522 3.426019
N 9.051140 0.527093 3.576665
H 9.004825 0.963614 4.525718
C 10.086204 -0.269606 3.148187
C 8.249638 0.767977 2.520105
H 7.365514 1.388767 2.578155
N 8.695627 0.160557 1.429091
C 9.845761 -0.489713 1.818384
H 10.431572 -1.079252 1.128361
C 10.327933 -3.085116 4.011283
O 11.213378 -3.772912 3.541913
C 8.548689 -0.153346 -3.172087
N 7.722876 -0.449220 -2.022379
C 6.587274 0.185929 -1.768724
O 5.907083 0.810182 -2.601433
H 8.092701 -1.003091 -1.255109
O 8.108420 1.948211 -0.602685
N 9.010528 2.977661 -0.570740
C 8.522156 4.021254 -1.122998
C 9.307506 5.295854 -1.082382
S 6.872108 3.958624 -1.793359
C 6.772615 5.574120 -2.616065
H 8.277978 -0.754436 -4.041769
H 9.584872 -0.369438 -2.915201
H 8.459795 0.907733 -3.415736
H 10.284595 5.074860 -0.653444
H 8.807875 6.031007 -0.444959
H 9.430624 5.753083 -2.065318
H 7.462265 5.644482 -3.457888
H 6.945316 6.405368 -1.933086
H 5.755621 5.646384 -3.003916
h 3.864915 -1.233180 -4.611490
h 6.434687 1.559161 -5.287910
h 2.197211 0.082800 0.696879
h 3.809767 2.386177 -1.609051
h 11.936140 -2.951652 6.440390
h 9.283732 -3.284418 3.770473

Reactant(SN3)

N	5.304177 -0.729951 -4.859783	C	9.078241 5.674032 -1.015191
H	5.563192 -0.439690 -3.924773	S	6.532043 5.516782 -2.314656
C	6.204244 -0.318276 -5.922369	C	7.044139 7.229820 -2.552247
H	5.944060 -0.806802 -6.862650	H	7.564874 -0.865352 -2.676324
H	7.188211 -0.717325 -5.637515	H	7.987506 -0.760518 -0.951465
C	6.479201 1.162733 -6.260080	H	9.264890 -0.782252 -2.211034
O	6.863636 1.392720 -7.394271	H	8.676687 6.315586 -0.227514
N	2.936480 0.779038 1.096394	H	9.535049 6.316734 -1.770941
H	2.891681 0.578300 2.094428	H	9.837153 5.023561 -0.581731
C	4.135088 1.492939 0.645445	H	7.305908 7.715057 -1.613180
H	4.461500 2.030482 1.542483	H	6.180894 7.735501 -2.983872
C	5.328665 0.615124 0.246050	H	7.865402 7.308947 -3.261551
H	5.468536 -0.143046 1.028774	h	4.318817 -0.660083 -5.015207
H	5.120978 0.079271 -0.694181	h	6.415962 1.964246 -5.524116
O	6.453152 1.441750 0.124647	h	2.456436 0.110893 0.527963
H	7.206888 1.003967 0.590235	h	3.892820 2.678648 -1.396504
C	3.742806 2.658385 -0.317082	h	11.765980 -2.894113 6.482735
O	3.135061 3.577444 0.216892	h	9.168330 -3.168178 3.679974
N	11.779241 -2.139614 5.826588		
H	12.702766 -1.848519 5.497284		
C	10.625460 -1.888986 4.973084		
H	9.769584 -1.571730 5.587171		
C	11.012774 -0.701721 4.048217		
H	11.452555 0.096205 4.651667		
H	11.780968 -1.062817 3.358030		
N	8.925074 0.671681 3.825444		
H	8.988779 1.065012 4.792001		
C	9.855048 -0.170806 3.268016		
C	8.005906 0.962308 2.883150		
H	7.159169 1.613763 3.056298		
N	8.277639 0.343699 1.745663		
C	9.429360 -0.368301 1.977017		
H	9.888421 -0.980204 1.214339		
C	10.197923 -3.043158 4.015195		
O	11.063546 -3.810181 3.639975		
C	8.263773 -0.435488 -1.960376		
N	8.220817 1.010946 -2.060097		
C	7.074606 1.637190 -2.293253		
O	6.040065 1.143136 -2.768616		
H	8.899357 1.557278 -1.545149		
O	7.028252 2.965887 -2.134238		
N	8.142893 3.551944 -1.546724		
C	7.994834 4.823455 -1.601688		

TS1(SN3)

N	5.328842	-0.741516	-4.758532
H	5.563753	-0.389612	-3.832302
C	6.235609	-0.326240	-5.817741
H	5.985267	-0.815428	-6.761838
H	7.220566	-0.718522	-5.523854
C	6.497731	1.158432	-6.141096
O	6.844621	1.398799	-7.287535
N	3.044148	0.727212	1.078286
H	3.023567	0.530001	2.077230
C	4.229040	1.422221	0.555965
H	4.623750	1.935431	1.439008
C	5.350985	0.477695	0.058748
H	5.501409	-0.274127	0.850879
H	5.028104	-0.062151	-0.840858
O	6.545622	1.156761	-0.182418
H	7.465834	0.772191	0.712978
C	3.777941	2.615631	-0.352901
O	3.189932	3.511944	0.237576
N	11.643988	-2.058793	5.718908
H	12.571265	-1.787900	5.379098
C	10.489134	-1.836059	4.851522
H	9.631475	-1.519889	5.462947
C	10.858402	-0.662469	3.896912
H	11.316399	0.142010	4.478713
H	11.609321	-1.033589	3.192828
N	8.811497	0.781818	3.710895
H	8.912011	1.142923	4.692895
C	9.692518	-0.105390	3.130198
C	7.892808	1.146203	2.809518
H	7.076587	1.831270	2.990427
N	8.131952	0.527163	1.659303
C	9.249504	-0.261988	1.840133
H	9.652858	-0.869779	1.045181
C	10.086102	-3.014777	3.912000
O	10.976749	-3.748988	3.531935
C	8.245236	-0.500018	-2.030953
N	8.177105	0.945846	-2.062440
C	6.919295	1.505162	-1.898221
O	5.931026	1.099222	-2.578894
H	8.869644	1.419587	-1.493700
O	6.929037	2.884138	-1.811709
N	8.061129	3.450494	-1.265361
C	7.993120	4.718582	-1.426824
C	9.116385	5.566892	-0.915423
S	6.564060	5.430897	-2.206699
C	7.155109	7.103876	-2.548796
H	7.514651	-0.912839	-2.727433
H	8.027598	-0.915117	-1.034271
H	9.235773	-0.819695	-2.357023
H	8.756113	6.279941	-0.168162
H	9.588577	6.140707	-1.717270
H	9.858156	4.917446	-0.449336
H	7.415435	7.649797	-1.641527
H	6.326773	7.614896	-3.041784
H	7.999969	7.103989	-3.237095
h	4.347739	-0.666314	-4.936726
h	6.456046	1.946474	-5.389219
h	2.497870	0.095342	0.528474
h	3.882679	2.654475	-1.437146
h	11.632570	-2.811912	6.376672
h	9.056822	-3.184632	3.596090

IM(SN3)

N	5.337808	-0.731587	-4.691834
H	5.549431	-0.326770	-3.778479
C	6.252167	-0.305422	-5.741252
H	6.013509	-0.783298	-6.693404
H	7.235829	-0.698403	-5.443861
C	6.523365	1.181005	-6.045128
O	6.867099	1.432681	-7.191028
N	3.269660	0.429635	1.029909
H	3.177924	0.384285	2.038783
C	4.329389	1.241118	0.437566
H	4.785877	1.736245	1.302218
C	5.418239	0.328189	-0.171949
H	5.617919	-0.451779	0.572467
H	5.061995	-0.158173	-1.082919
O	6.634288	0.992562	-0.424075
H	7.645677	0.757709	0.683577
C	3.764499	2.458236	-0.386140
O	3.108514	3.250104	0.276670
N	11.596934	-2.048322	5.674856
H	12.524983	-1.744179	5.366694
C	10.452611	-1.801545	4.804636
H	9.590458	-1.475839	5.406078
C	10.863300	-0.631851	3.865931
H	11.329540	0.161282	4.454895
H	11.612344	-1.021687	3.172394
N	8.835262	0.836627	3.648943
H	8.915519	1.189085	4.637978
C	9.725621	-0.051600	3.083510
C	7.939870	1.217070	2.737838
H	7.117850	1.899580	2.901897
N	8.215243	0.598189	1.594027
C	9.320727	-0.201438	1.784547
H	9.738893	-0.796499	0.988645
C	10.049575	-2.969367	3.848004
O	10.955941	-3.636942	3.392424
C	8.317211	-0.390162	-2.309147
N	8.184563	1.053077	-2.234694
C	6.887417	1.492836	-1.838174
O	5.910862	1.167880	-2.628763
H	8.885406	1.474971	-1.633993
O	6.878484	2.903603	-1.690563
N	8.003354	3.448089	-1.120608
C	7.978779	4.715230	-1.297062
C	9.113311	5.541999	-0.780450
S	6.584484	5.450953	-2.122480
C	7.233183	7.094723	-2.494679
H	7.590347	-0.786195	-3.020480
H	8.144546	-0.903103	-1.346938
H	9.311356	-0.641426	-2.680399
H	8.767405	6.274298	-0.046047
H	9.611278	6.090957	-1.582769
H	9.831189	4.879043	-0.299345
H	7.504146	7.654629	-1.600421
H	6.429287	7.620943	-3.008358
H	8.083380	7.048398	-3.173052
h	4.359795	-0.658682	-4.887148
h	6.491710	1.957071	-5.280415
h	2.579550	-0.075975	0.512146
h	3.865905	2.590994	-1.463247
h	11.574968	-2.801465	6.332326
h	9.014974	-3.192088	3.587114

TS2(SN3)

N	5.434754	-0.596516	-4.560776
H	5.631945	-0.208217	-3.644457
C	6.372789	-0.220998	-5.607881
H	6.118540	-0.695036	-6.558833
H	7.340591	-0.651064	-5.311829
C	6.668858	1.257580	-5.930019
O	6.951495	1.492038	-7.096659
N	3.594111	-0.181615	1.038444
H	3.371577	0.094177	1.993396
C	4.577050	0.662676	0.332482
H	5.213320	1.049131	1.138244
C	5.478016	-0.285198	-0.519538
H	5.478185	-1.242942	-0.001114
H	5.053374	-0.437553	-1.513833
O	6.843207	0.062017	-0.639475
H	7.583586	1.496929	0.844655
C	3.942713	1.968127	-0.298082
O	3.185955	2.570094	0.446837
N	11.496271	-1.969614	5.687361
H	12.422560	-1.687341	5.354378
C	10.342503	-1.736732	4.817973
H	9.480490	-1.437039	5.430773
C	10.720211	-0.544650	3.892347
H	11.225935	0.217279	4.493506
H	11.438244	-0.914216	3.154366
N	8.783225	1.036412	3.838463
H	8.917782	1.324630	4.839501
C	9.568361	0.111734	3.185611
C	7.901657	1.553952	2.981352
H	7.151806	2.292775	3.215861
N	8.066437	0.998372	1.791012
C	9.098805	0.090567	1.894951
H	9.419522	-0.499686	1.050621
C	9.960546	-2.902884	3.853202
O	10.876565	-3.565759	3.407982
C	8.895329	0.069173	-2.835851
N	8.564709	1.069034	-1.820613
C	7.190172	1.054366	-1.529276
O	6.378123	1.338475	-2.445646
H	9.095894	0.927188	-0.966969
O	7.204523	2.323226	-0.162104
N	8.297215	3.170267	-0.133383
C	8.113785	4.274069	-0.748442
C	9.208698	5.298937	-0.700792
S	6.580286	4.634721	-1.557415
C	6.946016	6.225792	-2.353438
H	8.568824	0.427370	-3.811803
H	8.439606	-0.912226	-2.642074
H	9.977845	-0.051152	-2.880049
H	8.863376	6.208363	-0.199393
H	9.558439	5.586608	-1.694210
H	10.038150	4.883173	-0.130406
H	7.155181	7.023118	-1.639341
H	6.046152	6.489903	-2.913192
H	7.771955	6.153792	-3.062314
h	4.460531	-0.611994	-4.785772
h	6.701016	2.046517	-5.178614
h	2.779752	-0.530221	0.574476
h	4.076119	2.286885	-1.331843
h	11.487562	-2.733638	6.332468
h	8.931154	-3.131235	3.577025

Product(SN3)

N	5.343868	-0.910543	-4.787139
H	5.587097	-0.647315	-3.840220
C	6.290115	-0.491931	-5.807691
H	6.022410	-0.914333	-6.777194
H	7.245240	-0.961074	-5.527874
C	6.637036	0.992666	-6.059667
O	6.953609	1.274026	-7.204276
N	3.474997	-0.271533	1.086550
H	3.281899	-0.005285	2.049921
C	4.529638	0.454344	0.374180
H	5.197589	0.811383	1.167549
C	5.330629	-0.612647	-0.418509
H	5.321511	-1.513793	0.190921
H	4.861590	-0.843857	-1.376677
O	6.699420	-0.320417	-0.610448
H	7.576405	1.535418	0.615907
C	4.018229	1.774705	-0.318752
O	3.329596	2.477189	0.402295
N	11.720840	-2.090885	5.888935
H	12.642059	-1.820442	5.534516
C	10.561796	-1.838168	5.039592
H	9.708637	-1.533418	5.662924
C	10.948068	-0.636790	4.136542
H	11.398646	0.142171	4.757144
H	11.713384	-0.986562	3.436332
N	8.927044	0.834230	3.944922
H	9.014562	1.205810	4.917637
C	9.795591	-0.062803	3.374797
C	8.017361	1.188066	3.016022
H	7.216420	1.892464	3.199358
N	8.231936	0.561746	1.872351
C	9.340503	-0.224799	2.088699
H	9.752373	-0.862761	1.319134
C	10.139548	-2.979474	4.063127
O	11.012954	-3.729926	3.671990
C	9.112263	0.863471	-2.925680
N	8.420641	0.226447	-1.836481
C	7.099190	0.255975	-1.731523
O	6.326280	0.715521	-2.594185
H	8.923126	-0.131394	-1.038212
O	7.280345	2.273327	0.013956
N	8.392569	3.074165	-0.053028
C	8.197666	4.131016	-0.742670
C	9.301947	5.142697	-0.844101
S	6.631458	4.432632	-1.519803
C	6.952736	5.973647	-2.427069
H	8.787010	1.902355	-3.028689
H	8.946806	0.330037	-3.864384
H	10.179190	0.834893	-2.712113
H	8.966546	6.119802	-0.485296
H	9.653007	5.272185	-1.870234
H	10.123073	4.804916	-0.213802
H	7.123528	6.823288	-1.764942
H	6.051381	6.168311	-3.011563
H	7.790160	5.884713	-3.120488
h	4.364532	-0.824093	-4.969898
h	6.679172	1.744437	-5.271548
h	2.663338	-0.612981	0.612636
h	4.154991	2.032213	-1.369015
h	11.717555	-2.862407	6.525107
h	9.111287	-3.109300	3.725650

Reactant(SN4)

N	4.988533 -0.203275 -4.441305	C	9.192537 6.147687 -0.910127
H	5.303943 0.092468 -3.526895	S	6.789409 6.129958 -2.459073
C	5.872723 0.076631 -5.553732	C	7.628369 7.664716 -2.904420
H	5.572639 -0.477665 -6.442744	H	8.368739 -0.002855 -3.167771
H	6.845300 -0.348292 -5.267052	H	7.056631 -0.186674 -1.977507
C	6.184757 1.515195 -6.019555	H	8.739261 -0.177137 -1.440678
O	6.577177 1.637667 -7.167897	H	8.837196 6.948482 -0.254857
N	2.663258 1.402347 1.414764	H	9.783440 6.607913 -1.704510
H	2.732577 1.065666 2.374575	H	9.824018 5.467144 -0.338261
C	3.874413 2.036697 0.874395	H	7.881897 8.272393 -2.036537
H	4.322424 2.520656 1.747567	H	6.920994 8.218956 -3.520850
C	4.955901 1.068054 0.371620	H	8.515941 7.473908 -3.506290
H	5.008366 0.221368 1.067493	h	3.999603 -0.105520 -4.552810
H	4.684709 0.653115 -0.610270	h	6.127657 2.377865 -5.355773
O	6.170732 1.770110 0.321177	h	2.070077 0.830706 0.847900
H	6.897342 1.171681 0.599597	h	3.659257 3.314019 -1.122951
C	3.512914 3.256111 -0.044389	h	11.607959 -2.987152 6.320808
O	2.951989 4.178543 0.533459	h	8.991687 -3.202515 3.506982
N	11.616880 -2.220742 5.678541		
H	12.536210 -1.935830 5.332997		
C	10.454567 -1.957294 4.841826		
H	9.602410 -1.655352 5.469548		
C	10.826081 -0.748145 3.942921		
H	11.266372 0.039418 4.558520		
H	11.591209 -1.085299 3.237519		
N	8.730470 0.628970 3.739863		
H	8.815988 1.049799 4.694608		
C	9.658803 -0.212735 3.179972		
C	7.787032 0.887789 2.814220		
H	6.938305 1.534634 2.997204		
N	8.040690 0.251830 1.679332		
C	9.214614 -0.432266 1.900788		
H	9.668483 -1.059446 1.146145		
C	10.011562 -3.106034 3.879290		
O	10.857616 -3.914212 3.549720		
C	8.039567 0.251399 -2.157927		
N	7.973118 1.686691 -1.954986		
C	6.819283 2.332079 -2.064607		
O	5.773640 1.879686 -2.556115		
H	8.694742 2.164841 -1.432219		
O	6.779712 3.624193 -1.716087		
N	7.960276 4.127894 -1.164664		
C	8.039082 5.371914 -1.463940		

TS1(SN4)

N 5.008069 -0.225770 -4.318079
H 5.296988 0.175450 -3.428259
C 5.894775 0.060776 -5.432479
H 5.600778 -0.497076 -6.323271
H 6.868801 -0.356596 -5.139773
C 6.194492 1.504990 -5.884205
O 6.547362 1.636954 -7.046589
N 2.797582 1.352903 1.383515
H 2.889648 1.016026 2.339534
C 3.977918 2.006134 0.783462
H 4.466585 2.480121 1.642112
C 5.012875 1.014402 0.191455
H 5.096988 0.183995 0.909837
H 4.640579 0.584592 -0.743601
O 6.277601 1.581102 -0.006538
H 7.240273 0.954559 0.764428
C 3.534664 3.242460 -0.085278
O 2.977816 4.134437 0.540743
N 11.494996 -2.136440 5.550446
H 12.416695 -1.847916 5.209894
C 10.326797 -1.901082 4.703939
H 9.475756 -1.602770 5.334265
C 10.670551 -0.700842 3.771831
H 11.142102 0.086435 4.366678
H 11.404903 -1.044710 3.037963
N 8.613123 0.736599 3.689755
H 8.747192 1.082245 4.675743
C 9.489090 -0.115120 3.050339
C 7.676932 1.140734 2.830251
H 6.859637 1.809478 3.058268
N 7.897852 0.585061 1.643244
C 9.029402 -0.204947 1.759906
H 9.423826 -0.772391 0.931263
C 9.908870 -3.072758 3.761217
O 10.780207 -3.849168 3.425303
C 8.017719 0.233511 -2.149122
N 7.920446 1.649345 -1.847026
C 6.650994 2.153878 -1.595135
O 5.680674 1.819790 -2.349473
H 8.631886 2.041123 -1.245641
O 6.655854 3.525099 -1.340310
N 7.857984 4.031764 -0.875488
C 7.980924 5.238428 -1.284629
C 9.179650 6.023520 -0.847522
S 6.721764 5.961962 -2.305167
C 7.558059 7.480005 -2.827729
H 8.194089 0.055473 -3.213239
H 7.100737 -0.288843 -1.860046
H 8.850121 -0.215567 -1.599826
H 8.885547 6.887326 -0.243147
H 9.749187 6.396973 -1.701909
H 9.820822 5.371996 -0.252247
H 7.831444 8.122717 -1.990627
H 6.839525 8.014658 -3.449961
H 8.435656 7.270612 -3.439671
h 4.023074 -0.126235 -4.458966
h 6.166068 2.359430 -5.208047
h 2.144507 0.821023 0.844471
h 3.636734 3.310530 -1.168336
h 11.493194 -2.897722 6.198843
h 8.886734 -3.218443 3.411817

IM(SN4)

N 4.947270 -0.284056 -4.169524
H 5.198793 0.275710 -3.349274
C 5.814552 -0.024232 -5.318951
H 5.480863 -0.568680 -6.203822
H 6.784484 -0.475941 -5.068275
C 6.141693 1.418143 -5.773880
O 6.524602 1.529030 -6.928723
N 2.765601 1.439291 1.334587
H 2.909666 1.089938 2.278193
C 3.907544 2.108613 0.679102
H 4.425946 2.599014 1.511245
C 4.914847 1.091853 0.079166
H 5.039421 0.305277 0.834977
H 4.511117 0.623407 -0.818148
O 6.198679 1.606552 -0.178097
H 7.356192 0.965005 0.770434
C 3.411363 3.333978 -0.188941
O 2.856264 4.212780 0.455114
N 11.387432 -2.088065 5.552188
H 12.313234 -1.805620 5.217632
C 10.234794 -1.845002 4.687822
H 9.375045 -1.536862 5.301022
C 10.624659 -0.655659 3.764222
H 11.116567 0.117600 4.360804
H 11.351924 -1.024825 3.035962
N 8.637662 0.875896 3.652515
H 8.772120 1.211063 4.643769
C 9.471524 -0.034177 3.034393
C 7.723170 1.311921 2.791495
H 6.929790 2.015027 2.997999
N 7.931087 0.707051 1.624611
C 9.015273 -0.138128 1.746429
H 9.366992 -0.738259 0.922011
C 9.833980 -3.009380 3.724795
O 10.735335 -3.719861 3.329464
C 7.899235 0.411557 -2.274492
N 7.773540 1.819364 -1.945691
C 6.456050 2.202429 -1.549185
O 5.504291 1.894138 -2.379345
H 8.442144 2.120828 -1.245561
O 6.429237 3.606249 -1.307707
N 7.607922 4.099686 -0.781598
C 7.801872 5.282190 -1.227894
C 9.010541 6.035332 -0.762094
S 6.618880 6.004603 -2.339736
C 7.533980 7.470600 -2.876397
H 7.480099 0.219119 -3.261285
H 7.393570 -0.252598 -1.559357
H 8.956878 0.144295 -2.315157
H 8.734419 6.923185 -0.185241
H 9.626124 6.361020 -1.604047
H 9.602169 5.369774 -0.132197
H 7.815731 8.121381 -2.048700
H 6.858031 8.023974 -3.529659
H 8.416152 7.205788 -3.459266
h 3.966211 -0.192359 -4.340070
h 6.105944 2.286984 -5.116687
h 2.086232 0.913613 0.822638
h 3.484832 3.394886 -1.274740
h 11.376307 -2.859421 6.188472
h 8.809329 -3.206041 3.409390

TS2(SN4)

N	4.854515	-0.168036	-4.144980
H	5.080946	0.395126	-3.325748
C	5.776941	0.035753	-5.265068
H	5.464296	-0.526791	-6.145651
H	6.727372	-0.421773	-4.961093
C	6.137778	1.456519	-5.749300
O	6.461292	1.543656	-6.922333
N	2.941317	1.148793	1.359267
H	3.087441	0.800367	2.302362
C	4.096017	1.711109	0.635771
H	4.762165	2.071566	1.427581
C	4.843872	0.576785	-0.146568
H	4.715497	-0.333266	0.438869
H	4.348016	0.420704	-1.103176
O	6.240092	0.656894	-0.366766
H	7.357726	2.013252	0.919729
C	3.638349	3.027747	-0.122225
O	3.063763	3.846631	0.573734
N	11.221436	-1.923145	5.537015
H	12.150762	-1.671522	5.192649
C	10.076217	-1.686404	4.651249
H	9.199922	-1.419694	5.258619
C	10.450586	-0.457378	3.770411
H	11.012564	0.243037	4.395796
H	11.126617	-0.801304	2.982270
N	8.639787	1.262413	3.879640
H	8.827483	1.486809	4.887783
C	9.312327	0.306574	3.153329
C	7.786737	1.907335	3.084197
H	7.120087	2.690671	3.405244
N	7.853896	1.409739	1.858599
C	8.797789	0.401763	1.883369
H	9.038689	-0.183316	1.008988
C	9.734126	-2.847936	3.664449
O	10.659658	-3.555899	3.320275
C	8.278148	0.455436	-2.853435
N	8.045789	1.294212	-1.685203
C	6.711789	1.505997	-1.352225
O	5.911383	1.870843	-2.250890
H	8.608273	1.051927	-0.879688
O	7.010309	2.801222	-0.039038
N	8.178455	3.524295	-0.151233
C	8.091968	4.554995	-0.899311
C	9.285542	5.460728	-0.966517
S	6.610095	4.938199	-1.786371
C	6.990764	6.600568	-2.403207
H	8.107808	1.030275	-3.764617
H	7.647534	-0.443314	-2.869888
H	9.319799	0.134810	-2.850082
H	9.044937	6.458912	-0.589294
H	9.655035	5.563375	-1.988354
H	10.065678	5.032960	-0.338725
H	7.135985	7.319577	-1.596936
H	6.115811	6.897965	-2.982723
H	7.857971	6.610226	-3.064101
h	3.877382	-0.122586	-4.352625
h	6.192883	2.332839	-5.103472
h	2.176159	0.718764	0.880121
h	3.738523	3.182651	-1.196486
h	11.199437	-2.698223	6.168474
h	8.728954	-3.055923	3.297790

Product(SN4)

N	4.857713	-0.461872	-4.626237
H	5.165628	-0.376830	-3.670051
C	5.825078	-0.099777	-5.643356
H	5.585936	-0.589993	-6.584314
H	6.766549	-0.555122	-5.307998
C	6.179753	1.357326	-6.002354
O	6.547238	1.538270	-7.151474
N	2.821049	1.046664	1.307858
H	2.951532	0.747065	2.270788
C	4.001305	1.514691	0.566462
H	4.688276	1.876187	1.340473
C	4.678918	0.312494	-0.155565
H	4.600560	-0.539036	0.520416
H	4.124269	0.064061	-1.058257
O	6.061772	0.405995	-0.452431
H	7.429236	1.845666	0.671337
C	3.616225	2.815662	-0.240698
O	3.117971	3.697754	0.435603
N	11.576160	-2.185445	5.715799
H	12.502202	-1.932567	5.362860
C	10.426074	-1.911581	4.859774
H	9.569871	-1.605919	5.479197
C	10.832583	-0.700121	3.979546
H	11.298728	0.053448	4.619852
H	11.591531	-1.045137	3.271267
N	8.837240	0.806650	3.821586
H	8.932845	1.160084	4.802077
C	9.696569	-0.083155	3.228721
C	7.948608	1.212693	2.896470
H	7.155126	1.917567	3.108258
N	8.169039	0.629473	1.728367
C	9.261594	-0.183716	1.929366
H	9.675954	-0.795272	1.140175
C	9.995430	-3.049405	3.881885
O	10.845851	-3.856564	3.561222
C	8.521378	1.324431	-2.869029
N	7.787006	0.715700	-1.777823
C	6.471774	0.875904	-1.634705
O	5.707946	1.341368	-2.493433
H	8.280050	0.472170	-0.927057
O	7.143679	2.671768	0.205139
N	8.332814	3.283531	-0.111397
C	8.147613	4.296218	-0.868350
C	9.313932	5.173597	-1.199254
S	6.519946	4.651953	-1.477503
C	6.788876	6.214288	-2.356368
H	8.113351	2.313717	-3.093125
H	8.488432	0.696711	-3.762557
H	9.558523	1.435436	-2.555902
H	9.120198	6.206873	-0.900204
H	9.540681	5.166461	-2.267975
H	10.173134	4.812510	-0.637127
H	7.079510	7.019754	-1.681572
H	5.821569	6.462936	-2.795485
H	7.519747	6.126629	-3.161595
h	3.891767	-0.223346	-4.726360
h	6.172246	2.180554	-5.287997
h	2.054534	0.601942	0.844555
h	3.688189	2.924997	-1.322795
h	11.555508	-2.960102	6.347821
h	8.982218	-3.150336	3.492938

Reactant(SN5)

N 5.711562 -0.575951 -4.319912
H 6.084537 -0.277490 -3.425276
C 6.520678 -0.232178 -5.488861
H 6.166393 -0.785818 -6.359576
H 7.515898 -0.645480 -5.275827
C 6.784398 1.208208 -5.963515
O 7.163522 1.307774 -7.117346
N 3.027781 1.423528 1.444972
H 3.093643 1.149090 2.422964
C 4.230058 2.029606 0.858110
H 4.672867 2.588713 1.689270
C 5.326689 1.035157 0.436060
H 5.393291 0.261117 1.213333
H 5.050536 0.528600 -0.503889
O 6.533052 1.725416 0.308138
H 7.240485 1.157703 0.703656
C 3.859133 3.165210 -0.161158
O 3.250154 4.104919 0.330326
N 11.791905 -2.100009 5.828016
H 12.701015 -1.854326 5.427933
C 10.609787 -1.893369 5.004430
H 9.766402 -1.575204 5.634958
C 10.965529 -0.724837 4.051923
H 11.419719 0.082539 4.630885
H 11.714702 -1.093866 3.345725
N 8.905677 0.692028 3.841961
H 9.022449 1.140282 4.779697
C 9.788116 -0.207410 3.297670
C 7.953051 0.949788 2.930893
H 7.137417 1.642506 3.101524
N 8.154997 0.252978 1.819132
C 9.305918 -0.469668 2.041142
H 9.719253 -1.137646 1.300141
C 10.180121 -3.094946 4.099849
O 11.059900 -3.849558 3.733831
C 8.547536 0.123766 -1.858707
N 8.455411 1.568121 -1.798032
C 7.282334 2.154384 -2.017936
O 6.286866 1.616774 -2.525923
H 9.106501 2.094693 -1.230945
O 7.176258 3.465305 -1.788750
N 8.312645 4.064064 -1.241837
C 8.304860 5.300815 -1.577584
C 9.401552 6.168496 -1.044909
S 7.014302 5.937228 -2.601583
C 7.750482 7.510475 -3.075797
H 7.773648 -0.336352 -1.238359
H 9.522297 -0.184040 -1.485094
H 8.443747 -0.229313 -2.886816
H 9.994406 6.604809 -1.850782
H 10.045345 5.567829 -0.402871
H 8.989157 6.994939 -0.461858
H 7.056708 7.971617 -3.776002
H 8.699819 7.372161 -3.590066
H 7.888577 8.180507 -2.229638
h 4.732833 -0.375696 -4.364169
h 6.691847 2.084036 -5.321311
h 2.450731 0.810674 0.905162
h 4.038968 3.151309 -1.236115
h 11.801599 -2.836878 6.503962
h 9.148601 -3.283164 3.802181

TS1(SN5)

N	5.683169 -0.580790 -4.214849
H	6.005460 -0.189681 -3.331964
C	6.514187 -0.238150 -5.368376
H	6.183320 -0.790304 -6.249734
H	7.506378 -0.646605 -5.132190
C	6.783536 1.205296 -5.827502
O	7.147967 1.307130 -6.987838
N	3.146846 1.401566 1.391784
H	3.253354 1.111187 2.360346
C	4.323882 2.009348 0.745032
H	4.811877 2.553174 1.562114
C	5.372613 0.987064 0.235395
H	5.446158 0.209319 1.018493
H	5.004246 0.488896 -0.671012
O	6.622235 1.553338 0.015898
H	7.498468 0.977176 0.880328
C	3.882048 3.162107 -0.231305
O	3.290745 4.086177 0.307678
N	11.683102 -2.014221 5.740695
H	12.594229 -1.780959 5.335802
C	10.500038 -1.832277 4.904761
H	9.655381 -1.516816 5.534005
C	10.843758 -0.670265 3.934041
H	11.331365 0.126877 4.502384
H	11.566989 -1.046924 3.204978
N	8.833449 0.820506 3.826617
H	8.985146 1.196226 4.796713
C	9.667355 -0.089249 3.213507
C	7.910623 1.233648 2.958585
H	7.128656 1.952094 3.167228
N	8.099118 0.616704 1.796039
C	9.189580 -0.218262 1.934817
H	9.546419 -0.834611 1.124192
C	10.096363 -3.049147 4.010044
O	10.998967 -3.768171 3.630275
C	8.478768 0.101268 -1.930049
N	8.356963 1.533484 -1.785198
C	7.073471 2.032212 -1.676291
O	6.135482 1.592701 -2.404176
H	9.003796 1.982383 -1.149443
O	7.021846 3.398556 -1.512664
N	8.191276 3.977110 -1.047793
C	8.238853 5.186994 -1.463009
C	9.390495 6.039855 -1.024473
S	6.942629 5.826177 -2.487857
C	7.706526 7.368116 -3.035393
H	7.775932 -0.435504 -1.279378
H	9.493550 -0.203884 -1.669795
H	8.297311 -0.204605 -2.963528
H	9.967910 6.409265 -1.875050
H	10.040490 5.448713 -0.378201
H	9.040386 6.914383 -0.469229
H	7.005361 7.823012 -3.734147
H	8.640208 7.193202 -3.569365
H	7.881446 8.066595 -2.217916
h	4.703637 -0.398093 -4.299180
h	6.712156 2.074615 -5.173853
h	2.516299 0.820292 0.877486
h	4.005744 3.151908 -1.314200
h	11.686759 -2.744999 6.423280
h	9.064988 -3.278196 3.741960

IM(SN5)

N	5.665150	-0.547021	-4.184823
H	5.960023	-0.096422	-3.316317
C	6.505643	-0.204269	-5.333371
H	6.195147	-0.769608	-6.213817
H	7.500386	-0.594702	-5.075497
C	6.766425	1.236661	-5.797945
O	7.118868	1.335074	-6.963169
N	3.229790	1.405998	1.333869
H	3.348251	1.145433	2.306041
C	4.358820	2.061471	0.654596
H	4.853304	2.616432	1.460612
C	5.398009	1.035754	0.138752
H	5.536080	0.306918	0.951145
H	5.017300	0.500085	-0.731521
O	6.662382	1.577891	-0.137179
H	7.651609	1.067939	0.891447
C	3.859704	3.206317	-0.308468
O	3.253475	4.102962	0.257376
N	11.637554	-2.010477	5.700563
H	12.550584	-1.746929	5.320319
C	10.461060	-1.800120	4.866411
H	9.615209	-1.475559	5.489719
C	10.846274	-0.642236	3.911882
H	11.358208	0.134703	4.485456
H	11.553725	-1.043303	3.181426
N	8.861309	0.885704	3.822426
H	9.003771	1.228776	4.808289
C	9.694196	-0.016570	3.195442
C	7.967681	1.354229	2.956221
H	7.190427	2.075347	3.170991
N	8.185644	0.772461	1.779792
C	9.253601	-0.089708	1.901523
H	9.607286	-0.682650	1.073235
C	10.054531	-3.006523	3.955311
O	10.966542	-3.675646	3.513948
C	8.482476	0.153447	-1.948057
N	8.311209	1.585600	-1.856826
C	6.982216	2.020795	-1.570133
O	6.054971	1.629722	-2.386602
H	8.961666	2.019819	-1.211347
O	6.957387	3.435569	-1.491786
N	8.120775	4.002991	-1.016554
C	8.194308	5.206817	-1.439784
C	9.366525	6.042383	-1.015895
S	6.901534	5.862579	-2.463393
C	7.710282	7.361244	-3.061825
H	8.048086	-0.404531	-1.102377
H	9.546532	-0.086113	-1.995724
H	8.029853	-0.227445	-2.865362
H	9.947571	6.387487	-1.873607
H	10.004698	5.440129	-0.368626
H	9.042750	6.929996	-0.466396
H	7.011307	7.831003	-3.752316
H	8.621443	7.137850	-3.615495
H	7.933371	8.071473	-2.267316
h	4.684719	-0.379009	-4.287293
h	6.699844	2.103381	-5.140351
h	2.561041	0.846657	0.844090
h	3.968289	3.204421	-1.393029
h	11.639374	-2.740421	6.384048
h	9.023378	-3.272944	3.723342

TS2(SN5)

N	5.685195 -0.521089 -4.181040	C	9.551353 5.463483 -0.899288
H	5.988504 -0.120081 -3.295888	S	6.926695 4.861634 -1.825876
C	6.521118 -0.174344 -5.328446	C	7.331170 6.472831 -2.554448
H	6.179781 -0.710430 -6.215561	H	8.245001 -0.593022 -2.612311
H	7.506199 -0.602612 -5.098529	H	9.865226 0.118182 -2.634863
C	6.801211 1.270050 -5.768560	H	8.598715 0.827850 -3.633516
O	7.117644 1.378200 -6.942899	H	9.951594 5.573851 -1.908092
N	3.363857 1.204837 1.393449	H	10.321367 5.062659 -0.241839
H	3.497004 0.921181 2.359094	H	9.277055 6.461329 -0.545546
C	4.530322 1.742723 0.665305	H	6.467668 6.736831 -3.167396
H	5.143949 2.203656 1.445793	H	8.208914 6.435981 -3.200404
C	5.315200 0.527392 0.046025	H	7.467927 7.251681 -1.803876
H	5.271956 -0.251402 0.809350	h	4.697818 -0.397620 -4.280104
H	4.738704 0.173160 -0.805804	h	6.787169 2.136686 -5.107645
O	6.691009 0.562670 -0.302917	h	2.623868 0.719831 0.927448
H	7.557595 2.028919 1.015289	h	4.141958 3.021389 -1.309257
C	4.050049 2.968523 -0.224442	h	11.402110 -2.526912 6.344589
O	3.460122 3.836307 0.392678	h	8.863242 -3.109396 3.676473
N	11.400035 -1.774382 5.686055		
H	12.315736 -1.563371 5.278289		
C	10.225783 -1.579769 4.827769		
H	9.367027 -1.302956 5.453967		
C	10.573322 -0.380966 3.900632		
H	11.146524 0.342253 4.489333		
H	11.233123 -0.752677 3.111264		
N	8.762034 1.339330 3.992903		
H	8.978149 1.598082 4.986099		
C	9.426333 0.365702 3.282110		
C	7.923913 1.990127 3.187771		
H	7.285700 2.807474 3.487293		
N	7.986626 1.467135 1.970511		
C	8.913902 0.444667 2.011763		
H	9.145134 -0.153577 1.144100		
C	9.881307 -2.782268 3.887652		
O	10.826858 -3.376718 3.409470		
C	8.801007 0.353100 -2.672270		
N	8.484131 1.274209 -1.588793		
C	7.124886 1.405551 -1.310942		
O	6.333540 1.655536 -2.253868		
H	9.030705 1.130929 -0.747669		
O	7.273860 2.788464 -0.035110		
N	8.437224 3.527001 -0.083530		
C	8.376118 4.534107 -0.866216		

Product(SN5)

N	5.675290 -0.737787 -4.347427	C	9.648141 5.241015 -1.059466
H	6.038052 -0.519965 -3.430285	S	6.900855 4.800586 -1.683683
C	6.525583 -0.338991 -5.464671	C	7.358998 6.293594 -2.604070
H	6.191937 -0.835454 -6.375147	H	8.789254 0.757626 -3.572339
H	7.505362 -0.780624 -5.236101	H	10.087187 1.296892 -2.509533
C	6.821117 1.118289 -5.854263	H	8.726746 2.392503 -2.863173
O	7.171422 1.261304 -7.013681	H	9.991070 5.181197 -2.093801
N	3.312309 1.130267 1.437349	H	10.424118 4.869194 -0.393649
H	3.425578 0.869234 2.411036	H	9.471729 6.293769 -0.826687
C	4.501987 1.611093 0.713416	H	6.490142 6.531216 -3.218302
H	5.129286 2.059079 1.491340	H	8.212216 6.139083 -3.264061
C	5.248248 0.355840 0.146847	H	7.556650 7.141130 -1.948607
H	5.256999 -0.358342 0.969836	h	4.699547 -0.523502 -4.391906
H	4.657262 -0.078158 -0.659328	h	6.781792 1.967170 -5.171679
O	6.615049 0.406954 -0.238725	h	2.574190 0.648141 0.965430
H	7.418488 2.198786 0.916657	h	4.179603 2.855420 -1.291668
C	4.090415 2.831043 -0.205612	h	11.525177 -2.594733 6.491614
O	3.542404 3.739060 0.391042	h	8.942538 -3.044586 3.785924
N	11.525869 -1.830465 5.846738		
H	12.433725 -1.643717 5.414630		
C	10.346241 -1.615444 5.005732		
H	9.495237 -1.331861 5.640159		
C	10.698594 -0.410137 4.094115		
H	11.238224 0.324301 4.698654		
H	11.387368 -0.769140 3.323698		
N	8.852867 1.266229 4.116400		
H	9.075845 1.603037 5.077529		
C	9.530247 0.274586 3.455436		
C	7.929211 1.778122 3.290626		
H	7.258313 2.578501 3.566524		
N	7.945430 1.164489 2.118894		
C	8.939397 0.215341 2.214593		
H	9.179372 -0.456889 1.401973		
C	9.969242 -2.793106 4.051822		
O	10.893135 -3.452635 3.616260		
C	9.015498 1.352154 -2.684260		
N	8.341288 0.809008 -1.528441		
C	7.014077 0.878322 -1.414853		
O	6.247564 1.241121 -2.321754		
H	8.847036 0.725690 -0.656788		
O	7.223877 2.910710 0.229007		
N	8.455415 3.479180 0.022643		
C	8.412657 4.428458 -0.833040		
